# Patient-reported outcomes between proton and photon therapy in nasopharyngeal carcinoma patients: A longitudinal cohort study

**DOI:** 10.1016/j.ctro.2025.100971

**Published:** 2025-05-02

**Authors:** Ching-Nung Wu, Yu-Ming Wang, Wei-Chih Chen, Yun-Hsuan Lin, Shau-Hsuan Li, Chung-Feng Hwang, Bing-Shen Huang, Chung-Ying Lin, Sheng-Dean Luo

**Affiliations:** a School of Traditional Chinese Medicine, Chang Gung University College of Medicine, Taoyuan, Taiwan; b Department of Otolaryngology, Kaohsiung Chang Gung Memorial Hospital and Chang Gung University College of Medicine, Kaohsiung, Taiwan; c Department of Otolaryngology, Kaohsiung Municipal Ta-Tung Hospital, Kaohsiung, Taiwan; d Department of Radiation Oncology, Kaohsiung Chang Gung Memorial Hospital and Chang Gung University College of Medicine, Kaohsiung, Taiwan; e Proton and Radiation Therapy Center, Kaohsiung Chang Gung Memorial Hospital and Chang Gung University College of Medicine, Kaohsiung, Taiwan; f School of Medicine, College of Medicine, National Sun Yat-sen University, Kaohsiung, Taiwan; g Department of Hematology-Oncology, Kaohsiung Chang Gung Memorial Hospital and Chang Gung University College of Medicine, Kaohsiung, Taiwan; h Department of Radiation Oncology and Proton and Radiation Therapy Center, Linkou Chang Gung Memorial Hospital, Taoyuan, Taiwan; i Graduate Institute of Clinical Medical Sciences, College of Medicine, Chang Gung University, Taoyuan, Taiwan; j Institute of Allied Health Sciences, College of Medicine, National Cheng Kung University, Tainan, Taiwan; k Department of Public Health, College of Medicine, National Cheng Kung University, Tainan, Taiwan; l Biostatistics Consulting Center, National Cheng Kung University Hospital, College of Medicine, National Cheng Kung University, Tainan, Taiwan; m School of Nursing, College of Nursing, Kaohsiung Medical University, Kaohsiung, Taiwan

**Keywords:** Nasopharyngeal carcinoma, Intensity-modulated proton therapy, Volumetric-modulated arc therapy, Symptom distress scale, Oral ulcer

## Abstract

**Background and purpose:**

This study aimed to compare patient-reported outcomes (PROs) between intensity-modulated proton therapy (IMPT) and volumetric-modulated arc therapy (VMAT) in treating nasopharyngeal carcinoma (NPC).

**Materials and methods:**

A prospective cohort study was conducted at a tertiary care center from 2021 to 2023. Newly diagnosed NPC patients were assessed using the modified 28-item symptom distress scale at seven time points, from baseline (T0) to one year post-radiotherapy (RT) (T6). The analysis focused on symptom changes during the acute phase (T0 to T3 [one month post-RT]) using linear mixed models. Sensitivity analyses incorporated inverse probability treatment weighting and data from non-metastatic patients adhering to survey protocols.

**Results:**

IMPT recipients (n = 65) had higher education levels, marital stability, household incomes, and better performance statuses compared to VMAT recipients (n = 36). Oral-related symptoms peaked by the seventh week of RT and declined thereafter. IMPT significantly reduced “oral ulcer” (coefficient: −0.83, SE: 0.27) and “difficulty opening mouth” (coefficient: −0.44, SE: 0.18) symptoms by the seventh week but did not improve “dry mouth.” “Tinnitus” was rated as more distressing than “hearing difficulty,” with no significant differences between IMPT and VMAT for ear-related symptoms. Sensitivity analyses validated these findings.

**Conclusion:**

IMPT was associated with reduced severity of specific oral-related symptoms during the acute phase of RT compared to VMAT. While these findings suggest potential benefits of IMPT, baseline differences between groups necessitate cautious interpretation and further validation through randomized studies.

## Highlights

• We tracked PROs at several time points to map symptom distress over IMPT versus VMAT.
• IMPT markedly reduced oral ulcer and difficulty opening mouth versus VMAT during acute RT, peaking at week 7.
• IMPT and VMAT showed similar xerostomia rates, highlighting need for further study.
• Tinnitus and hearing difficulty were similar, though IMPT patients had lower baseline ear distress.
• PROs are essential for comparing RT modalities’ effects on symptom management and QoL.

## Introduction

Radiotherapy (RT) with concurrent chemotherapy is a cornerstone in treating nasopharyngeal cancer (NPC) [1] Volumetric modulated arc therapy (VMAT), an advanced photon beam therapy, is widely utilized in managing NPC [2]. However, its effectiveness is sometimes compromised by the nasopharynx's close proximity to the cranial base, which can lead to troubling locoregional complications [3]. In contrast, intensity-modulated proton therapy (IMPT), a refined proton RT, is gaining favor for its superior clinical outcomes [4]. Comparative studies on these modalities are growing, highlighting differences in effectiveness and complications [5].

Proton beam RT minimizes radiation exposure to surrounding healthy tissues by concentrating energy on the tumor, ensuring superior dose decay and tissue protection. In contrast, conventional photon-based RT can deliver substantial radiation doses along its path, often leading to collateral tissue damage [6]. Despite the advancements in photon-based techniques like VMAT, it is associated with considerable acute and late side effects, such as grade 2–3 mucositis in 78 % of cases and xerostomia in 83 % [7]. On the other hand, IMPT has demonstrated a reduction in specific acute grade 2 or higher adverse events (AEs) by 25 %–66 % compared to VMAT in NPC patients [8,9]. These AEs were typically assessed using the Common Terminology Criteria for Adverse Events, monitored by clinicians [10]. However, patient-reported outcomes (PROs), directly reported by patients without clinician interpretation, are underrepresented in comparisons of IMPT and VMAT. Differences in quality of life (QoL) or self-perceived functional status [11] between the two treatments remain unclear. Incorporating patient perspectives provides a more holistic evaluation of treatment benefits [12] making PROs essential for assessing toxic effects. Evidence on how IMPT and VMAT impact PROs in NPC patients is needed to address this gap in the literature.

The primary aim of this prospective cohort study is to determine if patients treated with IMPT report lower levels of symptom distress compared to those undergoing VMAT, as assessed in a longitudinal survey at multiple time points, particularly during the acute phase of RT. Additionally, the study seeks to identify other factors that may influence patients' ratings of RT-related symptom distress.

## Methods

### Patient cohorts and data collection

This ongoing research project received ethical approval from the Ethical Review Committee of Kaohsiung Chang Gung Memorial Hospital (KCGMH), with approval numbers 201901691B0C601, 202100470B0, and 202200543B0C502. Informed consent was written and obtained from all participants before initiating the study. In this prospective study, the symptom distress scale (SDS; please see “Patient-reported outcomes – modified 28-item Symptom Distress Scale (SDS-m)” below for details) for patients treated with either proton or photon therapy for newly-diagnosed NPC at KCGMH from January 2021 to December 2023. Patients with recurrent NPC or significant comorbidities that could adversely affect QoL, such as end-stage renal disease (ESRD), hemiplegia, paraplegia, advanced liver cirrhosis, or other terminal-stage cancers, were excluded. Participants were surveyed at multiple time points: at baseline (T0), in the 4th week of RT (T1), in the 7th week of RT (T2), 1-month post-RT (T3), 3 months post-RT (T4), 6 months post-RT (T5), and 1-year post-RT (T6). Surveys were conducted in person, with the assistance of an experienced research assistant. We also collected data on patients’ demographics, socioeconomic status (SES), and clinical disease status during the survey. Only patients who completed at least the surveys at T0 and T1 were included for the final cohort analysis.

### Standard treatment protocol and radiation modalities – VMAT or IMPT

According to National Comprehensive Cancer Network (NCCN) guidelines, stage I NPC patients (AJCC 8th edition) received radiotherapy alone, while those with stage II to IVA underwent chemoradiotherapy [13]. Radiation doses to the gross tumor volume, high-risk, and low-risk sites were 69.96, 59.4, and 52.8 Gray equivalents, respectively, delivered over 33 daily sessions [14]. Concurrent chemoradiotherapy included weekly cisplatin (40 mg/m^2^) for up to seven weeks. For stage II to IVA cases, induction chemotherapy was added following NCCN guidelines.

IMPT plans were developed using the RayStation system from RaySearch Laboratories in Stockholm, Sweden, utilizing the pencil beam line scanning technology. Most patients were treated with three beams, incorporating robust optimization that accounted for 3 mm of setup uncertainties and 3.5 percent range uncertainties. We employed two anterior oblique beams and one posterior-anterior beam, which allowed for effective dose distribution management to sensitive structures. Treatment goals aimed for 99.5 % clinical target volume coverage while minimizing exposure to nearby organs at risk [15]. For VMAT, plans used at least two arcs, with a 3 mm planning target volume margin added to manage uncertainties. Optimization ensured 95 % of the planning target volume received 100 % of the prescribed dose [15]. In Taiwan, VMAT is reimbursed by national health insurance, minimizing costs for patients, while IMPT requires significant out-of-pocket expenses. Radiation oncologists discuss the benefits and limitations of both options with patients to support informed decision-making.

### Patient-reported outcomes – modfied 28-item symptom distress scale (SDS-m)

Symptom distress was assessed using a modified Chinese version of the 28-item Symptom Distress Scale (SDS), which evolved from the modified 24-item SDS and the original 13-item SDS [16,17]. The 24-item SDS is a Likert-type scale, with responses ranging from 1 (indicating no distress) to 5 (indicating extreme distress). This scale has been validated as reliable in cancer-related research across Mandarin-speaking regions [18,19], exhibiting a Cronbach’s alpha between 0.80 and 0.91 [16]. To better capture otorhinologic symptoms associated with RT in NPC patients, we expanded the scale by including four additional items based on clinical experiences and literature review: [20,21] tinnitus, nasal blockage, neck hyperpigmentation, and neck stiffness, thereby creating the modified 28-item SDS (SDS-m).

To evaluate the differential impact of IMPT versus VMAT on local otolaryngological symptoms in NPC treatment, we specifically selected symptoms and grouped them into two categories. The first group includes oral-related symptoms such as dry mouth, oral ulcers, and difficulty opening the mouth. The second group encompasses ear-related symptoms, including tinnitus and hearing difficulties. We analyzed the changes in symptom scores from each time point relative to baseline (e.g., T1 minus T0, T2 minus T0, etc.), with a particular focus on symptom score changes during the acute phase of radiotherapy (i.e., from baseline to 1-month post-radiotherapy, T0 to T3).

### Covariates

The survey captured a range of demographic and clinical variables, including age, gender, education level, marital status, annual household income, and the Karnofsky Performance Status (KPS) at baseline (T0). Additionally, disease-specific information such as clinical staging, based on the AJCC 8th edition TNM staging system, and details regarding the chemotherapy regimen were obtained through chart reviews. This study was designed to investigate the association between different RT modalities and RT-related changes in the SDS. According to the causal directed acyclic graphs [22], potential confounders—variables that could influence both the choice of RT modalities and SDS outcomes—included the aforementioned covariates (Fig. S1). It is important to note that performance and disease status at SDS assessment time points beyond T0 were considered intermediate variables in the relationship between RT and the SDS changes, rather than as confounders.

### Statistical analysis

To evaluate differences between groups (IMPT *vs*. VMAT), the chi-square test or Fisher's exact test was used for categorical covariates, while the Student’s *t*-test or Mann–Whitney *U* test was employed for continuous variables, depending on whether they were parametric or nonparametric. Linear mixed models, with participants as random factors, were utilized to explore factors influencing changes in the SDS across repeated assessments during the acute phase of RT. These models included confounders to construct the multivariable regression models. Additionally, we considered the interaction between RT modalities and specific time points within the acute RT phase concerning changes in the SDS. Within these models, positive coefficients suggested a prediction of worsening RT-related symptoms. To determine clinical significance, effect sizes were calculated using Cohen's d (small: 0.2, medium: 0.5, large: 0.8), owing to the lack of established minimal clinically important differences (MCID) for the assessment instruments among NPC patients [23].

To enhance the robustness of our findings and address potential confounding between the groups, the inverse probability of treatment weighting (IPTW) method was employed [24]. This involved calculating the likelihood of undergoing either IMPT or VMAT based on individual characteristics such as age, gender, education level, household income, marital status, KPS, AJCC 8th edition staging, and chemotherapy regimen (i.e., propensity score). We then derived weights as the inverse of these propensity scores and applied them within the linear mixed models to further equalize the groups. A sensitivity analysis was conducted among non-metastatic NPC patients who completed all four SDS-m assessments during the acute phase of radiotherapy (from T0 to T3). Statistical analyses were performed using R version 4.2.1 and the Statistical Analysis System software version 9.4 (SAS Institute, Cary, NC, USA). All p-values reported were two-sided.

## Results

One hundred and ten patients were screened for their eligibility for the present study: eight declined to participate, and one was excluded due to missing T1 survey data. Therefore, a total of 101 participants who received either VMAT or IMPT as their primary treatment were enrolled in the study for data analysis. Table 1 presents the characteristics of the participants, divided by the type of therapeutic intervention they received. Those who underwent IMPT generally had higher levels of education, were more likely to be married, and had higher household incomes. Although not statistically significant, a better performance status was observed among this group. In terms of disease severity, a higher proportion of patients treated with VMAT had T4 and stage IV disease, yet there were no significant differences in overall disease status or the use of induction chemotherapy and concurrent chemoradiotherapy between the two groups. Among the 101 participants analyzed, none experienced disease recurrence or death during the study period. However, at the T3 time point, four patients initiated UFUR maintenance therapy—three due to distant metastasis and one due to pre-existing N3 disease before treatment. These included three patients in the IMPT group (3/65, 4.6 %) and one in the VMAT group (1/36, 2.8 %). Table S1 shows the descriptive statistics of the 28-item SDS-m across seven time points in this longitudinal study. A high rate of follow-up was achieved during the acute phase of RT (96 % or 97 out of 101), and 59 of the 101 participants (58.4 %) completed the one-year longitudinal follow-up.

**Table 1:** Baseline characteristics of patients stratified by radiation modality.

Participant characteristics	VMAT (n = 36)	IMPT (n = 65)	*p* value^a^
Age at RT, median (IQR); years	52.0 (41.0–58.5)	49.0 (43.0–58.0)	0.82
Gender; *n* (%)			0.55
Female	9 (25.0)	13 (20.0)	
Male	27 (75.0)	52 (80.0)	
Education level; *n* (%)			<0.01
High School Diploma or Less	28 (77.8)	21 (32.3)	
College Degree or Higher	8 (22.2)	44 (67.7)	
Marital status; *n* (%)			0.02
Single	9 (25.0)	12 (18.5)	
Married	19 (52.8)	49 (75.4)	
Divorced/Separated/Widowed	8 (22.2)	4 (6.1)	
Yearly Household Income ($); *n* (%)			<0.01
<20,000	24 (66.7)	16 (24.6)	
20000–33333	7 (19.4)	15 (23.1)	
>33,333	5 (13.9)	34 (52.3)	
Karnofsky Performance Status			0.14
100	9 (25.0)	28 (43.1)	
90	24 (66.7)	35 (53.8)	
80	3 (8.3)	2 (3.1)	
Tumor status; *n* (%)			0.24
T1	13 (36.1)	30 (46.2)	
T2	5 (13.9)	13 (20.0)	
T3	8 (22.2)	14 (21.5)	
T4	10 (27.8)	8 (12.3)	
Nodal status; *n* (%)			0.94
N0	6 (16.7)	12 (18.5)	
N1	9 (25.0)	18 (27.7)	
N2	12 (33.3)	22 (33.8)	
N3	9 (25.0)	13 (20.0)	
Clinical AJCC staging; *n* (%)			0.62
I	4 (11.1)	7 (10.8)	
II	5 (13.9)	9 (13.9)	
III	10 (27.8)	27 (41.5)	
IVa	15 (41.7)	18 (27.7)	
IVb	2 (5.5)	4 (6.1)	
Induction chemotherapy; *n* (%)	33 (91.7)	57 (87.7)	0.74
Treatment regimen; *n* (%)			1
RT alone	3 (8.3)	6 (9.2)	
Chemoradiotherapy	33 (91.7)	59 (90.8)	

Abbreviations: AJCC, American Joint Committee on Cancer; IMPT, Intensity-modulated proton therapy; RT, Radiotherapy; VMAT, Volumetric Modulated Arc Therapy.

a Categorical variables were assessed by χ2 test or Fisher exact test. Parametric continuous variable was compared by Student’s *t*-test.

**Fig. 1:**
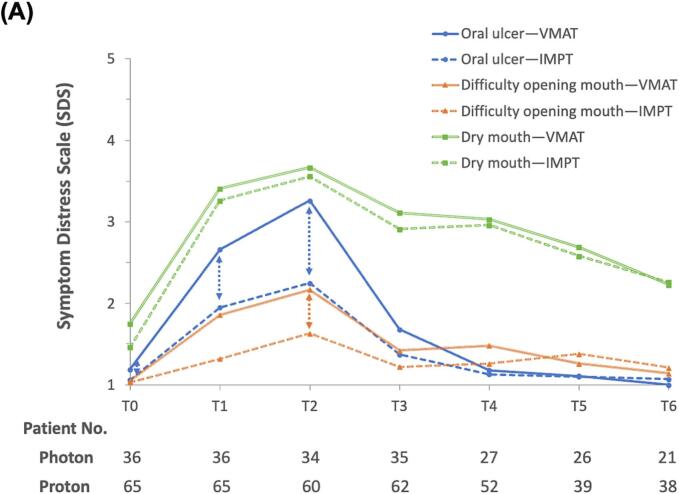

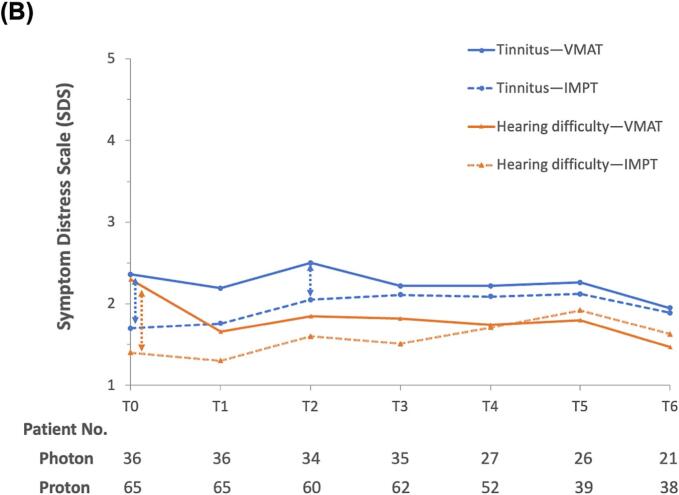
Longitudinal changes in the mean symptom distress scale scores among nasopharyngeal carcinoma patients treated with either intensity-modulated proton therapy (IMPT) or volumetric-modulated arc therapy (VMAT) within one year. (A) Oral-related symptoms. (B) Ear-related symptoms. Time points: Baseline (T0), 4th week of radiotherapy (RT) (T1), 7th week of RT (T2), 1-month post-RT (T3), 3 months post-RT (T4), 6 months post-RT (T5), and 1-year post-RT (T6). Note: A dashed line with double arrows indicates statistically significant differences between the RT techniques at specific clinical time points.

The longitudinal trend for oral-related symptoms indicated that distress levels increased by the 4th week of radiotherapy (T1), peaked in the 7th week (T2), and then declined from the 1st month post-radiotherapy (T3) through to the 1st year post-radiotherapy (T6), as illustrated in Fig. 1a. During the acute phase of RT, patients treated with IMPT demonstrated significant improvements in “oral ulcer” symptoms in both unadjusted analyses (coef.: −0.49, SE: 0.18, *p*-value < 0.01) and analyses adjusted for multiple variables (coef.: −0.49, SE: 0.21, *p*-value = 0.02) compared to those receiving VMAT. The Cohen’s d value was 0.43, indicating a medium-size effect. A similar benefit was observed in the symptom “difficulty opening mouth”, although the effect size was reduced in both the unadjusted (coef.: −0.38, SE: 0.12, *p-*value < 0.01) and multivariable analyses (coef.: −0.27, SE: 0.13, *p-*value = 0.04). The Cohen's d value was 0.48, which also indicates a medium-size effect size. For “dry mouth,” IMPT did not exhibit a better outcome. Other covariates had minimal impact on oral-related symptoms, except for the baseline symptom score. A poor baseline SDS status was associated with less worsening of distress during the acute phase of RT compared to those with a better baseline SDS status (Table 2).

**Table 2:** Regression coefficients for change of oral related symptom distress scale within acute phase of radiotherapy derived from mixed model analyses.

Participant characteristics	Dry mouth		Oral ulcer		Difficulty opening mouth	
	Crude	Multivariable	Crude	Multivariable	Crude	Multivariable
Treatment modalities (*IMPT vs. VMAT*)	0.16 (0.23)	−0.11 (0.23)	*−0.49 (0.18)	*−0.49 (0.21)	*−0.38 (0.12)	*−0.27 (0.13)
Baseline symptoms score	*−0.73 (0.10)	*−0.82 (0.12)	*−0.78 (0.29)	*−1.20 (0.30)	*−0.68 (0.30)	*−0.68 (0.29)
Age *(per 10-year increase)*	−0.00 (0.10)	0.03 (0.11)	−0.01 (0.08)	−0.10 (0.10)	−0.09 (0.06)	*−0.21 (0.06)
Gender (*ref: female*)	−0.08 (0.26)	−0.36 (0.24)	−0.32 (0.21)	−0.29 (0.21)	*−0.31 (0.14)	−0.18 (0.13)
Education (*ref: high school diploma or less*)	0.12 (0.22)	0.11 (0.25)	−0.35 (0.18)	−0.23 (0.22)	−0.19 (0.12)	−0.17 (0.14)
Marital status (*ref: single*)						
Married	0.28 (0.28)	−0.12 (0.30)	0.07 (0.23)	0.14 (0.26)	0.24 (0.15)	* 0.43 (0.17)
Divorced/Separated/Widowed	0.45 (0.41)	−0.05 (0.40)	−0.01 (0.34)	−0.34 (0.35)	0.39 (0.22)	0.34 (0.23)
Yearly Household income($) (*ref: <20000*)						
20000–33333	0.04 (0.30)	−0.14 (0.29)	0.02 (0.24)	0.13 (0.26)	0.07 (0.16)	−0.00 (0.16)
>33,333	0.17 (0.25)	0.01 (0.27)	−0.37 (0.20)	−0.08 (0.24)	−0.22 (0.14)	−0.20 (0.15)
Karnofsky Performance Status (*ref: 100*)						
90	−0.03 (0.23)	0.04 (0.20)	0.21 (0.19)	0.26 (0.18)	0.13 (0.13)	0.11 (0.12)
80	−0.23 (0.52)	0.36 (0.47)	0.73 (0.42)	* 0.84 (0.42)	0.41 (0.28)	0.19 (0.27)
Tumor status (*ref: T1*)						
T2	−0.33 (0.30)	−0.25 (0.27)	−0.04 (0.25)		−0.18 (0.17)	
T3	*−0.70 (0.28)	−0.49 (0.28)	0.30 (0.24)		0.14 (0.16)	
T4	−0.44 (0.31)	−0.63 (0.36)	0.10 (0.26)		−0.10 (0.18)	
Nodal status (*ref: N0*)						
N1	−0.40 (0.33)		0.04 (0.28)		0.19 (0.18)	
N2	−0.36 (0.32)		−0.06 (0.27)		0.22 (0.18)	
N3	−0.32 (0.35)		0.08 (0.29)		0.01 (0.19)	
Clinical AJCC staging (*ref: I*)						
II	−0.31 (0.44)	0.36 (0.39)	0.06 (0.36)	0.34 (0.34)	0.23 (0.24)	0.20 (0.21)
III	−0.59 (0.37)	0.12 (0.37)	0.15 (0.31)	0.20 (0.29)	0.34 (0.21)	0.30 (0.19)
IVa	−0.45 (0.38)	0.17 (0.38)	0.23 (0.32)	0.21 (0.29)	0.16 (0.21)	0.13 (0.19)
IVb	−0.80 (0.55)	−0.36 (0.49)	−0.32 (0.46)	−0.41 (0.42)	0.03 (0.31)	−0.09 (0.27)
Induction chemotherapy	−0.41 (0.36)		0.11 (0.30)		0.24 (0.20)	
Chemoradiotherapy (*ref: RT alone*)	−0.58 (0.37)		0.25 (0.31)		0.25 (0.21)	

Abbreviations: AJCC, American Joint Committee on Cancer; IMPT, Intensity-modulated proton therapy; RT, Radiotherapy; VMAT, Volumetric Modulated Arc Therapy.

*p ≤ 0.05.

In examining the longitudinal trend of ear-related symptoms, the shift from T0 to T6 was negligible, even during the acute phase of RT. Patients undergoing VMAT reported significantly higher baseline distress scores compared to those receiving IMPT. Moreover, “tinnitus” was reported to be more troublesome than “hearing difficulty” among ear-related symptoms (Fig. 1b). In terms of changes in ear-related symptoms during the acute phase of RT, there were no significant differences between the groups treated with IMPT and VMAT. However, patients with higher educational levels were significantly associated with less distress from tinnitus (coef.: −0.54, SE: 0.25, *p*-value = 0.02), whereas those with poorer KPS scores experienced greater distress (coef.: 0.48, SE: 0.20, *p*-value = 0.01). Additionally, a poor baseline SDS status was linked to less exacerbation of distress during the acute phase of RT compared to those with a better baseline SDS status (Table 3).

**Table 3:** Regression coefficients for change of otologic related symptom distress scale within acute phase of radiotherapy derived from mixed model analyses.

Participant characteristics	Tinnitus		Hearing difficulty	
	Crude	Multivariable	Crude	Multivariable
Treatment modalities (*IMPT vs. VMAT*)	0.43 (0.26)	0.04 (0.24)	*0.58 (0.25)	−0.07 (0.19)
Baseline symptoms score	*−0.75 (0.08)	*−0.81 (0.09)	*−0.84 (0.07)	*−0.92 (0.08)
Age *(per 10-year increase)*	−0.10 (0.12)	−0.21 (0.11)	0.08 (0.11)	0.09 (0.09)
Gender (*ref: female*)	0.16 (0.30)	−0.01 (0.23)	0.26 (0.29)	−0.02 (0.18)
Education (*ref: high school diploma or less*)	0.10 (0.25)	*−0.54 (0.25)	−0.03 (0.24)	−0.34 (0.20)
Marital status (*ref: single*)				
Married	−0.25 (0.32)	−0.15 (0.29)	−0.11 (0.30)	−0.43 (0.23)
Divorced/Separated/Widowed	−0.25 (0.47)	−0.02 (0.39)	−0.35 (0.45)	−0.23 (0.31)
Yearly household income($) (*ref: < 20000*)				
20000–33333	0.12 (0.34)	−0.23 (0.28)	−0.15 (0.32)	−0.10 (0.23)
>33,333	0.31 (0.29)	0.29 (0.27)	0.20 (0.27)	0.25 (0.21)
Karnofsky Performance Status (*ref: 100*)				
90	0.20 (0.26)	0.64 (0.50)	0.05 (0.25)	0.30 (0.16)
80	−1.08 (0.59)	* 0.48 (0.20)	*−1.14 (0.56)	0.64 (0.39)
Tumor status (*ref: T1*)				
T2	−0.02 (0.35)		0.04 (0.33)	
T3	−0.33 (0.33)		−0.35 (0.31)	
T4	−0.46 (0.37)		−0.54 (0.35)	
Nodal status (*ref: N0*)				
N1	−0.16 (0.38)		0.09 (0.37)	
N2	0.41 (0.37)		−0.08 (0.35)	
N3	0.10 (0.40)		−0.03 (0.39)	
Clinical AJCC staging (*ref: I*)				
II	−0.59 (0.50)	−0.22 (0.37)	−0.31 (0.48)	0.11 (0.30)
III	−0.05 (0.43)	−0.04 (0.32)	−0.42 (0.41)	0.03 (0.26)
IVa	−0.30 (0.44)	−0.33 (0.32)	−0.54 (0.42)	0.04 (0.26)
IVb	−0.95 (0.63)	−0.85 (0.47)	−0.50 (0.61)	−0.30 (0.38)
Induction chemotherapy	−0.03 (0.42)		−0.21 (0.40)	
Chemoradiotherapy (*ref: RT alone*)	−0.38 (0.44)		−0.46 (0.41)	

Abbreviations: AJCC, American Joint Committee on Cancer; IMPT, Intensity-modulated proton therapy; RT, Radiotherapy; VMAT, Volumetric Modulated Arc Therapy.

*p ≤ 0.05.

Concerning the impact of IMPT on SDS changes at different time points within the acute phase of RT, interaction effects were explored (Table 4). Specifically, although patients treated with IMPT experienced significant reductions in SDS for “oral ulcer” and “difficulty opening mouth” during the acute phase, the most notable improvements were observed in the 7th week of RT (coef.: −0.83, SE: 0.27, *p*-value < 0.01, for “oral ulcer”; coef.: −0.44, SE: 0.18, *p*-value = 0.01, for “difficulty opening mouth”). Furthermore, when IPTW method was employed in an alternative analysis (Table S2), these effects were even more pronounced and were evident as early as the 4th week of radiotherapy (coef.: −0.75, SE: 0.23, *p*-value < 0.01, for “oral ulcer”; coef.: −0.40, SE: 0.15, *p*-value = 0.01, for “difficulty opening mouth”). Additionally, a sensitivity analysis was conducted with 86 non-metastatic NPC patients who completed the first four consecutive surveys during the acute phase of RT (Table S3). The findings from this analysis were consistent with those from the main analysis (Table S4).

**Table 4:** Interaction effects of radiation modality and time points on symptom distress change via multivariable regression and IPTW models.

Group and Interaction effect	Multivariable regression	IPTW with regression
Dry mouth		
Acute phase within RT *(IMPT vs. VMAT)*	−0.11 (0.23)	−0.04 (0.18)
4th week during RT *(IMPT x T1*^a^)	−0.14 (0.27)	−0.15 (0.22)
7th week during RT *(IMPT x T2*^b^)	−0.08 (0.27)	−0.04 (0.23)
1 month after RT *(IMPT x T3*^c^)	−0.11 (0.27)	0.06 (0.23)
Oral ulcer		
Acute phase within RT *(IMPT vs. VMAT)*	*−0.49 (0.21)	*−0.56 (0.17)
4th week during RT *(IMPT x T1*^a^)	−0.51 (0.27)	*−0.75 (0.23)
7th week during RT *(IMPT x T2*^b^)	*−0.83 (0.27)	*−0.77 (0.23)
1 month after RT *(IMPT x T3*^c^)	−0.13 (0.27)	−0.19 (0.23)
Difficulty opening mouth		
Acute phase within RT *(IMPT vs. VMAT)*	*−0.27 (0.13)	*−0.34 (0.11)
4th week during RT *(IMPT x T1*^a^)	−0.32 (0.18)	*−0.40 (0.15)
7th week during RT *(IMPT x T2*^b^*)*	*−0.44 (0.18)	*−0.41 (0.16)
1 month after RT *(IMPT x T3*^c^*)*	−0.07 (0.18)	−0.22 (0.16)
Tinnitus		
Acute phase within RT *(IMPT vs. VMAT)*	0.04 (0.24)	−0.13 (0.20)
4th week during RT *(IMPT x T1*^a^*)*	−0.03 (0.26)	−0.26 (0.23)
7th week during RT *(IMPT x T2*^b^*)*	−0.04 (0.27)	−0.28 (0.23)
1 month after RT *(IMPT x T3*^c^*)*	0.19 (0.26)	0.15 (0.23)
Hearing difficulty		
Acute phase within RT *(IMPT vs. VMAT)*	−0.07 (0.19)	−0.21 (0.17)
4th week during RT *(IMPT x T1*^a^*)*	−0.12 (0.23)	−0.26 (0.20)
7th week during RT *(IMPT x T2*^b^*)*	−0.00 (0.23)	−0.18 (0.20)
1 month after RT *(IMPT x T3*^c^*)*	−0.08 (0.23)	−0.19 (0.20)

Abbreviations: IMPT, Intensity-modulated proton therapy; IPTW, inverse probability of treatment weighting; RT, Radiotherapy; VMAT, Volumetric Modulated Arc Therapy.

a T1 refers to 4th week during RT.

b T2 refers to 7th week during RT.

c T3 refers to 1 month after RT.

## Discussion

To the best of our knowledge, this research is among the pioneering efforts to assess symptom distress using PROs by patients undergoing IMPT, in comparison to those receiving VMAT for the treatment of NPC in a longitudinal framework. Our results indicate that IMPT significantly alleviates symptoms such as “oral ulcer” and “difficulty opening mouth” during the acute phase of RT. Furthermore, we observed that patients with lower educational attainment and those with diminished performance status reported heightened distress related to tinnitus.

IMPT has been associated with fewer oral-related AEs in NPC treatment, primarily due to its ability to minimize radiation to normal tissues like the salivary glands and larynx [25]. Li X et al. reported reduced rates of dysphagia, dysgeusia, mucositis, and weight loss with IMPT [8], while Chou et al. found lower rates of nasogastric tube insertion and weight loss, aligning with our findings on “oral ulcer” and “difficulty opening mouth [9]. An analysis of our previous report showed that the mean dose to the oral cavity in IMPT was 14.59 ± 3.52 Gy (RBE), whereas in VMAT, it was 34.15 ± 3.21 Gy (RBE), with a statistically significant difference (p < 0.001), further supporting our current findings [15]. Additionally, our study detailed the progression of distress across the acute phase of RT in a one-year longitudinal cohort. We specifically examined the interaction between RT modalities and time points within this phase, assessing changes in the SDS. This analysis helped us understand more precisely when the differences in symptoms due to various RT modalities occur. Overall, the benefits of IMPT began from the 4th week of radiotherapy, with the most significant improvements observed in the 7th week for both ”oral ulcer“ and ”difficulty opening mouth.“

Surprisingly, our study found no significant difference in dry mouth (xerostomia) between IMPT and VMAT patients, revealing inconsistencies in the literature about IMPT's benefits. For instance, Li X et al. found that patients treated with IMPT had reduced rates of at least grade 2 xerostomia (7.1 %) compared to those receiving IMRT (22.4 %), suggesting some advantage of IMPT over IMRT in this aspect [8]. However, Chou YC et al. observed no statistically significant difference in rates of xerostomia between patients treated with IMPT and those treated with VMAT (11.3 % *vs*. 16.3 %) [9]. Additionally, studies on organs at risk (OARs) showed no significant dose reduction to salivary glands with IMPT compared to IMRT or VMAT [26,27]. It highlights the need for more detailed studies to truly understand IMPT's role in reducing xerostomia, as patient experiences and quantitative dose measurements might tell different stories about this symptom's management. While IMPT offers dosimetric advantages in reducing oral cavity toxicity, data on acute skin toxicity with proton therapy are mixed. Several studies [9,28,29], have reported higher rates of grade 3 radiation dermatitis following IMPT, whereas Li et al. [8] found no significant difference compared to photon-based IMRT. In our cohort, patient-reported appearance distress (SDS Appearance item) did not differ between IMPT and IMRT (Table S1). These inconsistencies underscore the need for further prospective studies to clarify the true incidence and risk factors of acute radiation dermatitis in IMPT.

NPC patients often experience persistent otological complications, such as otitis media with effusion (OME), radiation-induced otitis media, and sensorineural hearing loss, despite advancements in photon-based therapies like IMRT or VMAT [30]. These symptoms remain prevalent even with advanced RT techniques [21]. Our study examined whether IMPT, with its precise energy deposition via the Bragg peak, could improve ear symptoms. While IMRT or VMAT typically delivers 40–50 Gy to the cochlea, IMPT reduces this to 30–40 Gy [26,27] However, no significant reduction in ear symptoms was observed with IMPT, potentially due to the timing of our assessments. Functional impairments like Eustachian tube dysfunction often manifest around 6 months post-RT and improve over 5 years [31]. Our one-year post-RT timeframe may have been insufficient to capture long-term differences. Interestingly, while no short-term benefits in ear symptoms were observed with IMPT, patients receiving IMPT reported less otologic distress before the start of RT. This could be because patients with higher SES are more vigilant about their health and tend to seek medical advice earlier, leading to earlier diagnoses and potentially better initial health status [32].

Several limitations were present in our study. First, only approximately 60 % of participants completed surveys at all seven time points. Nevertheless, the majority of differences in patient-rated symptom distress among treatment modalities were observed within the acute phase of RT (Table S1), where the survey completion rate was 96 %. This high follow-up rate within this phase helped minimize potential selection bias in our cohort study. Observations beyond the acute phase were not part of the main analysis but were monitored for trend analysis. Second, the timing of the baseline survey may be a concern, as a subset of patients (13 out of 101, 12.9 %) had pre-induction chemotherapy PRO scores (T(−)), including 3 out of 36 (8.3 %) in the VMAT group and 10 out of 65 (15.4 %) in the IMPT group. However, the chi-square test (*p* = 0.48) indicated no significant difference in the proportion of T(−) cases between the two groups, suggesting a minimal confounding effect. Additionally, a comparison of T0 and T(−) scores for the five primary SDS domains—dry mouth, oral ulcer, difficulty opening mouth, tinnitus, and hearing difficulty—showed no statistically significant differences, further supporting the limited impact of using either T0 or T(−) as the baseline survey time point. While this finding was somewhat unexpected, it reinforces the robustness of our analytical approach. Third, differences in the characteristics of populations opting for IMPT versus VMAT could raise concerns. In Taiwan, VMAT treatments are covered by NHI, whereas IMPT is not, leading to higher out-of-pocket expenses for those choosing IMPT. This inherent bias is challenging to address without a randomized controlled trial. To help mitigate this issue, SES, such as household income and education level, was included as a proxy variable in our regression analysis (Fig. S1). Additionally, IPTW was used to balance the characteristics between the two treatment groups and further bridge this research gap. Lastly, the absence of established reference scores for clinical significance when comparing group averages on various items is a limitation. It is crucial to understand that statistical differences do not automatically translate to clinical significance and vice versa, due to factors like small sample sizes or the non-random selection of participants. Therefore, we used Cohen's d to calculate effect sizes, aiming to establish a foundation for determining the MCID for NPC patients in future studies.

## Conclusion

In conclusion, IMPT demonstrated a reduction in the severity of specific oral-related symptoms during the acute phase of RT compared with VMAT. This reduction was associated with a significantly lower Dmean to the oral cavity in IMPT-treated patients, supporting its dosimetric advantage in reducing radiation exposure to critical structures and mitigating treatment-related symptom distress. While these results suggest potential advantages of IMPT in mitigating certain patient-reported symptoms during NPC treatment, it is important to interpret these findings with caution given the baseline differences between groups. Further studies, ideally randomized controlled trials, are warranted to validate these observations and better understand the timing and magnitude of IMPT's benefits in improving patient care and QoL.

## CRediT authorship contribution statement

**Ching-Nung Wu:** Funding acquisition, Conceptualization, Writing – original draft. **Yu-Ming Wang:** Investigation. **Wei-Chih Chen:** Visualization. **Yun-Hsuan Lin:** Resources. **Shau-Hsuan Li:** Visualization. **Chung-Feng Hwang:** Data curation. **Bing-Shen Huang:** Data curation. **Chung-Ying Lin:** Writing – review & editing, Supervision. **Sheng-Dean Luo:** Project administration, Conceptualization.

## Declaration of generative AI and AI-assisted technologies in the writing process

During the preparation of this work the authors used ChatGPT4 in order to improve language and readability. After using this tool/service, the authors reviewed and edited the content as needed and takes full responsibility for the content of the publication.

## Funding

This study was partially funded by 10.13039/100012553Chang Gung Memorial Hospital Research Program under grants CORPG8M0321. It is essential to emphasize that the funders were not involved in any aspect of the study, including the design, data collection, analysis, decision to publish, or preparation of the manuscript. Their contribution was purely financial and had no bearing on the scientific integrity or the presentation of the study results.

## Declaration of competing interest

The authors declare that they have no known competing financial interests or personal relationships that could have appeared to influence the work reported in this paper.
