# Supplementary material for: Patient-reported outcomes between proton and photon therapy in nasopharyngeal carcinoma patients: A longitudinal cohort study
Source: Clin Transl Radiat Oncol. 2025 May 2;53:100971. doi: 10.1016/j.ctro.2025.100971 (PMC12223559; doi:10.1016/j.ctro.2025.100971)
Supplement: Supplementary Data 1 [file mmc1.docx]

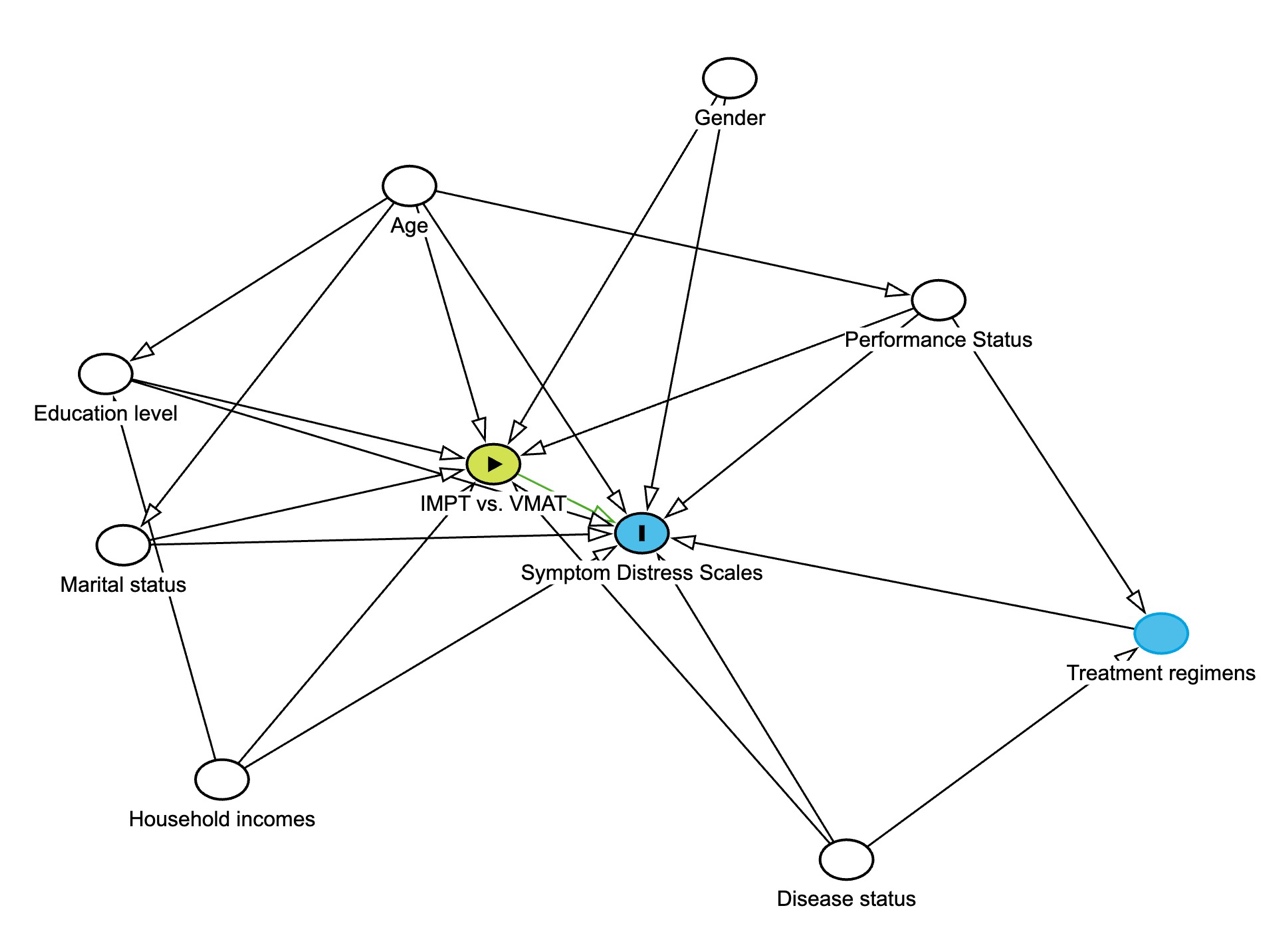


**Figure S1.** Directed acyclic graph of the associations between radiation modality and symptoms distress scales. IMPT, Intensity-modulated proton therapy; VMAT, volumetric-modulated arc therapy. The white colored circles represent confounders that were controlled for, while the blue colored circles represent variables that were not necessarily conditioned on in our study.

**Table S1.** Descriptive statistics summarized participants' 28-item symptom distress scale related with RT in seven measured time points.

| **Symptoms** | **Timing ^a^** | T0 | T1 | T2 | T3 | T4 | T5 | T6 |
| --- | --- | --- | --- | --- | --- | --- | --- | --- |
|  | **Number (Photon)** | 36 | 36 | 34 | 35 | 27 | 26 | 21 |
|  | **Number (Proton)** | 65 | 65 | 60 | 62 | 52 | 39 | 38 |
| Nausea | Photon |  |  |  |  |  |  |  |
|  | Mean (SD) | 1.25 (0.60) | 2.61 (1.22) | 2.23 (1.15) | 1.48 (0.74) | 1.14 (0.60) | 1.07 (0.27) | 1.09 (0.30) |
|  | Median (IQR) | 1 (0) | 2 (1.5) | 2 (2) | 1 (1) | 1 (0) | 1 (0) | 1 (0) |
|  | Proton |  |  |  |  |  |  |  |
|  | Mean (SD) | 1.16 (0.48) | 2.44 (1.1) | 2.20 (1.13) | 1.30 (0.58) | 1.17 (0.51) | 1.12 (0.33) | 1.07 (0.35) |
|  | Median (IQR) | 1 (0) | 2 (1) | 2 (1) | 1 (0) | 1 (1) | 1 (1) | 1 (1) |
|  | *p*-value | 0.37 | 0.57 | 0.89 | 0.27 | 0.46 | 0.52 | 0.57 |
| Vomiting | Photon |  |  |  |  |  |  |  |
|  | Mean (SD) | 1.19 (0.57) | 2.22 (1.39) | 2.08 (1.23) | 1.31 (0.58) | 1.03 (0.19) | 1.07 (0.27) | 1.00 (0.00) |
|  | Median (IQR) | 1 (0) | 2 (2) | 2 (2) | 1 (1) | 1 (0) | 1 (0) | 1 (0) |
|  | Proton |  |  |  |  |  |  |  |
|  | Mean (SD) | 1.06 (0.29) | 1.81 (1.05) | 1.75 (1.05) | 1.17 (0.38) | 1.05 (0.23) | 1.07 (0.26) | 1.05 (0.22) |
|  | Median (IQR) | 1 (0) | 1 (2) | 1 (1) | 1 (0) | 1 (0) | 1 (0) | 1 (0) |
|  | *p*-value | 0.10 | 0.18 | 0.17 | 0.29 | 0.70 | 1.00 | 0.30 |
| Loss of appetite | Photon |  |  |  |  |  |  |  |
|  | Mean (SD) | 1.52 (0.81) | 3.41 (1.33) | 3.61 (1.23) | 2.17 (1.09) | 2 (0.96) | 1.46 (0.85) | 1.28 (0.56) |
|  | Median (IQR) | 1 (1) | 4 (3) | 4 (2) | 2 (2) | 2 (2) | 1 (1) | 1 (0) |
|  | Proton |  |  |  |  |  |  |  |
|  | Mean (SD) | 1.38 (0.76) | 3.29 (1.08) | 3.48 (1.08) | 2.08 (1.02) | 1.61 (0.69) | 1.41 (0.63) | 1.10 (0.31) |
|  | Median (IQR) | 1 (1) | 3 (1) | 3 (1) | 2 (2) | 2 (1) | 1 (1) | 1 (0) |
|  | *p*-value | 0.38 | 0.54 | 0.47 | 0.74 | 0.08 | 0.88 | 0.16 |
| Insomnia | Photon |  |  |  |  |  |  |  |
|  | Mean (SD) | 2.05 (1.09) | 2.11 (1.06) | 2.73 (1.35) | 1.97 (0.89) | 1.81 (0.96) | 1.69 (0.97) | 1.38 (0.66) |
|  | Median (IQR) | 2 (2) | 2 (2) | 3 (2) | 2 (2) | 1 (2) | 1 (1) | 1 (1) |
|  | Proton |  |  |  |  |  |  |  |
|  | Mean (SD) | 1.81 (1.14) | 2.12 (1.08) | 2.2 (1.17) | 1.72 (0.79) | 1.73 (0.84) | 1.74 (0.96) | 1.78 (0.84) |
|  | Median (IQR) | 1 (1) | 2 (2) | 2 (2) | 2 (1) | 2 (1) | 1 (1) | 2 (1) |
|  | *p*-value | 0.18 | 0.99 | 0.06 | 0.17 | 0.84 | 0.79 | *0.03 |
| Pain | Photon |  |  |  |  |  |  |  |
|  | Mean (SD) | 1.69 (1.00) | 2.25 (1.13) | 2.64 (1.36) | 1.60 (0.77) | 1.33 (0.73) | 1.42 (0.70) | 1.28 (0.56) |
|  | Median (IQR) | 1 (1) | 2 (2) | 2.5 (3) | 1 (1) | 1 (0) | 1 (1) | 1 (0) |
|  | Proton |  |  |  |  |  |  |  |
|  | Mean (SD) | 1.53 (0.86) | 1.93 (0.98) | 2.30 (1.18) | 1.48 (0.56) | 1.42 (0.66) | 1.48 (0.75) | 1.21 (0.52) |
|  | Median (IQR) | 1 (1) | 2 (1) | 2 (2) | 1 (1) | 1 (1) | 1 (1) | 1 (0) |
|  | *p*-value | 0.49 | 0.16 | 0.25 | 0.71 | 0.32 | 0.72 | 0.49 |
| Fatigue | Photon |  |  |  |  |  |  |  |
|  | Mean (SD) | 1.77 (0.83) | 2.75 (1.25) | 2.82 (1.14) | 2.00 (0.93) | 1.74 (0.85) | 1.65 (0.84) | 1.61 (0.66) |
|  | Median (IQR) | 2 (1) | 2.5 (2) | 2.5 (2) | 2 (2) | 2 (1) | 1 (1) | 2 (1) |
|  | Proton |  |  |  |  |  |  |  |
|  | Mean (SD) | 1.84 (0.93) | 2.67 (0.95) | 2.56 (0.96) | 1.98 (0.89) | 1.94 (0.84) | 1.84 (0.77) | 1.86 (0.96) |
|  | Median (IQR) | 2 (1) | 3 (1) | 3 (1) | 2 (2) | 2 (1) | 2 (1) | 2 (1) |
|  | *p*-value | 0.85 | 0.89 | 0.49 | 0.97 | 0.25 | 0.24 | 0.43 |
| Bowel disturbance | Photon |  |  |  |  |  |  |  |
|  | Mean (SD) | 1.19 (0.46) | 2.19 (1.03) | 2.41 (1.25) | 1.88 (0.99) | 1.77 (1.05) | 1.57 (0.90) | 1.61 (1.02) |
|  | Median (IQR) | 1 (0) | 2 (2) | 2 (2) | 2 (2) | 1 (1) | 1 (1) | 1 (1) |
|  | Proton |  |  |  |  |  |  |  |
|  | Mean (SD) | 1.38 (0.76) | 2.04 (1.11) | 2.01 (1.15) | 1.43 (0.64) | 1.26 (0.48) | 1.23 (0.42) | 1.21 (0.47) |
|  | Median (IQR) | 1 (1) | 2 (2) | 2 (1.5) | 1 (1) | 1 (0.5) | 1 (0) | 1 (0) |
|  | *p*-value | 0.24 | 0.39 | 0.10 | *0.02 | *0.01 | 0.16 | 0.13 |
| Urinary problems | Photon |  |  |  |  |  |  |  |
|  | Mean (SD) | 1.19 (0.46) | 1.33 (0.63) | 1.50 (0.78) | 1.20 (0.47) | 1.18 (0.48) | 1.23 (0.51) | 1.00 (0.00) |
|  | Median (IQR) | 1 (0) | 1 (0.5) | 1 (1) | 1 (0) | 1 (0) | 1 (0) | 1 (0) |
|  | Proton |  |  |  |  |  |  |  |
|  | Mean (SD) | 1.18 (0.60) | 1.47 (0.75) | 1.33 (0.68) | 1.29 (0.58) | 1.3 (0.61) | 1.35 (0.66) | 1.21 (0.62) |
|  | Median (IQR) | 1 (0) | 1 (1) | 1 (0) | 1 (0) | 1 (0.5) | 1 (1) | 1 (0) |
|  | *p*-value | 0.44 | 0.31 | 0.22 | 0.43 | 0.31 | 0.42 | 0.08 |
| Dyspnea | Photon |  |  |  |  |  |  |  |
|  | Mean (SD) | 1.61 (1.04) | 1.19 (0.52) | 1.64 (0.94) | 1.28 (0.51) | 1.29 (0.77) | 1.19 (0.49) | 1.23 (0.53) |
|  | Median (IQR) | 1 (1) | 1 (0) | 1 (1) | 1 (1) | 1 (0) | 1 (0) | 1 (0) |
|  | Proton |  |  |  |  |  |  |  |
|  | Mean (SD) | 1.10 (1.04) | 1.27 (1.04) | 1.18 (1.04) | 1.14 (1.04) | 1.09 (1.04) | 1.10 (1.04) | 1.13 (1.04) |
|  | Median (IQR) | 1 (0) | 1 (0) | 1 (0) | 1 (0) | 1 (0) | 1 (0) | 1 (0) |
|  | *p*-value | *<0.01 | 0.45 | *<0.01 | *0.04 | 0.41 | 0.34 | 0.51 |
| Cough | Photon |  |  |  |  |  |  |  |
|  | Mean (SD) | 1.52 (0.65) | 1.77 (0.92) | 2.29 (1.26) | 1.71 (0.82) | 1.40 (0.74) | 1.30 (0.54) | 1.38 (0.49) |
|  | Median (IQR) | 1 (1) | 2 (1) | 2 (2) | 1 (1) | 1 (1) | 1 (1) | 1 (1) |
|  | Proton |  |  |  |  |  |  |  |
|  | Mean (SD) | 1.24 (0.66) | 1.69 (0.76) | 1.58 (0.78) | 1.37 (0.65) | 1.21 (0.45) | 1.17 (0.45) | 1.15 (0.36) |
|  | Median (IQR) | 1 (0) | 2 (1) | 1 (1) | 1 (1) | 1 (0) | 1 (0) | 1 (0) |
|  | *p*-value | *<0.01 | 0.80 | *<0.01 | *0.03 | 0.26 | 0.26 | 0.05 |
| Abdominal distention | Photon |  |  |  |  |  |  |  |
|  | Mean (SD) | 1.25 (0.60) | 1.61 (0.83) | 1.82 (1.05) | 1.28 (0.57) | 1.18 (0.48) | 1.07 (0.27) | 1.23 (0.53) |
|  | Median (IQR) | 1 (0) | 1 (1) | 1 (2) | 1 (0) | 1 (0) | 1 (0) | 1 (0) |
|  | Proton |  |  |  |  |  |  |  |
|  | Mean (SD) | 1.35 (0.75) | 1.96 (0.99) | 1.65 (0.84) | 1.19 (0.43) | 1.07 (0.26) | 1.15 (0.36) | 1.15 (0.54) |
|  | Median (IQR) | 1 (0) | 2 (2) | 1 (1) | 1 (0) | 1 (0) | 1 (0) | 1 (0) |
|  | *p*-value | 0.61 | 0.07 | 0.69 | 0.49 | 0.30 | 0.36 | 0.37 |
| Dry mouth | Photon |  |  |  |  |  |  |  |
|  | Mean (SD) | 1.75 (0.93) | 3.41 (1.10) | 3.67 (1.06) | 3.11 (1.23) | 3.03 (1.15) | 2.69 (1.22) | 2.23 (1.09) |
|  | Median (IQR) | 1 (1) | 3 (1) | 4 (2) | 3 (2) | 3 (2) | 2 (2) | 2 (1) |
|  | Proton |  |  |  |  |  |  |  |
|  | Mean (SD) | 1.46 (0.83) | 3.26 (1.00) | 3.56 (1.16) | 2.91 (1.10) | 2.96 (0.92) | 2.58 (1.09) | 2.26 (0.97) |
|  | Median (IQR) | 1 (1) | 3 (1) | 4 (1) | 3 (2) | 3 (1) | 2 (1) | 2 (1) |
|  | *p*-value | 0.08 | 0.51 | 0.80 | 0.41 | 0.71 | 0.80 | 0.81 |
| Oral ulcer | Photon |  |  |  |  |  |  |  |
|  | Mean (SD) | 1.19 (0.40) | 2.66 (1.49) | 3.26 (1.44) | 1.68 (0.93) | 1.18 (0.62) | 1.11 (0.43) | 1.00 (0.00) |
|  | Median (IQR) | 1 (0) | 3 (3) | 3.5 (3) | 1 (1) | 1 (0) | 1 (0) | 1 (0) |
|  | Proton |  |  |  |  |  |  |  |
|  | Mean (SD) | 1.06 (0.24) | 1.95 (1.16) | 2.25 (1.18) | 1.37 (0.68) | 1.13 (0.34) | 1.1 (0.38) | 1.07 (0.35) |
|  | Median (IQR) | 1 (0) | 2 (2) | 2 (2) | 1 (1) | 1 (0) | 1 (0) | 1 (0) |
|  | *p*-value | *0.04 | *0.02 | *<0.01 | 0.08 | 0.82 | 1.00 | 0.30 |
| Restlessness | Photon |  |  |  |  |  |  |  |
|  | Mean (SD) | 1.75 (0.80) | 1.94 (1.16) | 2.05 (1.17) | 1.57 (0.91) | 1.25 (0.59) | 1.19 (0.49) | 1.19 (0.51) |
|  | Median (IQR) | 2 (1) | 1 (2) | 2 (2) | 1 (1) | 1 (0) | 1 (0) | 1 (0) |
|  | Proton |  |  |  |  |  |  |  |
|  | Mean (SD) | 1.78 (1.06) | 1.83 (0.91) | 1.71 (0.86) | 1.46 (0.69) | 1.44 (0.72) | 1.35 (0.70) | 1.34 (0.62) |
|  | Median (IQR) | 1 (1) | 2 (1) | 2 (1) | 1 (1) | 1 (1) | 1 (1) | 1 (1) |
|  | *p*-value | 0.66 | 0.94 | 0.24 | 0.81 | 0.20 | 0.32 | 0.30 |
| Concentration | Photon |  |  |  |  |  |  |  |
|  | Mean (SD) | 1.44 (0.60) | 2.05 (1.11) | 2 (1.10) | 1.48 (0.81) | 1.55 (0.69) | 1.30 (0.54) | 1.19 (0.51) |
|  | Median (IQR) | 1 (1) | 2 (2) | 2 (2) | 1 (1) | 1 (1) | 1 (1) | 1 (0) |
|  | Proton |  |  |  |  |  |  |  |
|  | Mean (SD) | 1.47 (0.86) | 1.86 (0.93) | 1.8 (0.83) | 1.5 (0.71) | 1.4 (0.63) | 1.43 (0.75) | 1.36 (0.71) |
|  | Median (IQR) | 1 (1) | 2 (1) | 2 (1) | 1 (1) | 1 (1) | 1 (1) | 1 (1) |
|  | *p*-value | 0.54 | 0.49 | 0.54 | 0.76 | 0.32 | 0.64 | 0.29 |
| Appearance | Photon |  |  |  |  |  |  |  |
|  | Mean (SD) | 1.33 (0.82) | 1.75 (1.02) | 2.14 (1.25) | 1.54 (0.81) | 1.33 (0.73) | 1.00 (0.00) | 1.14 (0.47) |
|  | Median (IQR) | 1 (0) | 1 (1) | 2 (2) | 1 (1) | 1 (0) | 1 (0) | 1 (0) |
|  | Proton |  |  |  |  |  |  |  |
|  | Mean (SD) | 1.21 (0.64) | 1.61 (0.84) | 2.18 (0.91) | 1.66 (0.72) | 1.32 (0.55) | 1.33 (0.62) | 1.15 (0.43) |
|  | Median (IQR) | 1 (0) | 1 (1) | 2 (1) | 2 (1) | 1 (1) | 1 (1) | 1 (0) |
|  | *p*-value | 0.44 | 0.64 | 0.41 | 0.25 | 0.62 | *<0.01 | 0.72 |
| Bleeding | Photon |  |  |  |  |  |  |  |
|  | Mean (SD) | 1.50 (0.77) | 1.52 (0.84) | 1.55 (0.82) | 1.14 (0.42) | 1.07 (0.38) | 1.03 (0.19) | 1.00 (0.00) |
|  | Median (IQR) | 1 (1) | 1 (1) | 1 (1) | 1 (0) | 1 (0) | 1 (0) | 1 (0) |
|  | Proton |  |  |  |  |  |  |  |
|  | Mean (SD) | 1.38 (0.72) | 1.12 (0.41) | 1.31 (0.72) | 1.17 (0.46) | 1.07 (0.26) | 1.15 (0.36) | 1.02 (0.16) |
|  | Median (IQR) | 1 (1) | 1 (0) | 1 (0) | 1 (0) | 1 (0) | 1 (0) | 1 (0) |
|  | *p*-value | 0.38 | *<0.01 | 0.09 | 0.67 | 0.53 | 0.14 | 0.47 |
| Chills | Photon |  |  |  |  |  |  |  |
|  | Mean (SD) | 1.19 (0.46) | 2.05 (1.19) | 2.11 (1.32) | 1.57 (0.81) | 1.55 (0.93) | 1.30 (0.73) | 1.09 (0.30) |
|  | Median (IQR) | 1 (0) | 2 (2) | 2 (3) | 1 (1) | 1 (1) | 1 (0) | 1 (0) |
|  | Proton |  |  |  |  |  |  |  |
|  | Mean (SD) | 1.21 (0.64) | 1.4 (0.82) | 1.58 (0.88) | 1.25 (0.51) | 1.17 (0.43) | 1.17 (0.50) | 1.13 (0.47) |
|  | Median (IQR) | 1 (0) | 1 (0) | 1 (1) | 1 (0) | 1 (0) | 1 (0) | 1 (0) |
|  | *p*-value | 0.62 | *<0.01 | *0.04 | *0.04 | *0.04 | 0.48 | 0.89 |
| Fever | Photon |  |  |  |  |  |  |  |
|  | Mean (SD) | 1.05 (0.23) | 1.13 (0.35) | 1.05 (0.23) | 1.00 (0.00) | 1.03 (0.19) | 1.00 (0.00) | 1.00 (0.00) |
|  | Median (IQR) | 1 (0) | 1 (0) | 1 (0) | 1 (0) | 1 (0) | 1 (0) | 1 (0) |
|  | Proton |  |  |  |  |  |  |  |
|  | Mean (SD) | 1.04 (0.27) | 1.04 (0.21) | 1.06 (0.31) | 1.03 (0.17) | 1.01 (0.13) | 1.05 (0.22) | 1.05 (0.32) |
|  | Median (IQR) | 1 (0) | 1 (0) | 1 (0) | 1 (0) | 1 (0) | 1 (0) | 1 (0) |
|  | *p*-value | 0.56 | 0.10 | 0.87 | 0.29 | 0.64 | 0.25 | 0.47 |
| Numbness | Photon |  |  |  |  |  |  |  |
|  | Mean (SD) | 1.22 (0.42) | 1.36 (0.79) | 1.47 (0.92) | 1.34 (0.68) | 1.7 (1.03) | 1.69 (0.88) | 1.52 (0.67) |
|  | Median (IQR) | 1 (0) | 1 (0) | 1 (1) | 1 (0) | 1 (1) | 1 (1) | 1 (1) |
|  | Proton |  |  |  |  |  |  |  |
|  | Mean (SD) | 1.06 (0.29) | 1.38 (0.65) | 1.38 (0.71) | 1.59 (0.89) | 1.76 (1.09) | 1.48 (0.72) | 1.42 (0.85) |
|  | Median (IQR) | 1 (0) | 1 (1) | 1 (1) | 1 (1) | 1 (1) | 1 (1) | 1 (0) |
|  | *p*-value | *<0.01 | 0.45 | 0.92 | 0.14 | 0.94 | 0.38 | 0.23 |
| Tightness in chest | Photon |  |  |  |  |  |  |  |
|  | Mean (SD) | 1.33 (0.67) | 1.50 (0.77) | 1.35 (0.59) | 1.20 (0.40) | 1.22 (0.50) | 1.19 (0.49) | 1.19 (0.40) |
|  | Median (IQR) | 1 (0.5) | 1(1) | 1(1) | 1(0) | 1(0) | 1(0) | 1(0) |
|  | Proton |  |  |  |  |  |  |  |
|  | Mean (SD) | 1.18 (0.49) | 1.29 (0.57) | 1.33 (0.54) | 1.12 (0.38) | 1.11 (0.32) | 1.20 (0.52) | 1.13 (0.47) |
|  | Median (IQR) | 1 (0) | 1 (0) | 1 (1) | 1 (0) | 1 (0) | 1 (0) | 1 (0) |
|  | *p*-value | 0.17 | 0.11 | 0.98 | 0.26 | 0.37 | 0.99 | 0.25 |
| Burning sensation in the stomach | Photon |  |  |  |  |  |  |  |
|  | Mean (SD) | 1.25 (0.64) | 2.19 (1.16) | 1.97 (1.05) | 1.28 (0.51) | 1.25 (0.65) | 1.30 (0.67) | 1.33 (0.57) |
|  | Median (IQR) | 1 (0) | 2 (2) | 2 (1) | 1 (1) | 1 (0) | 1 (0) | 1 (1) |
|  | Proton |  |  |  |  |  |  |  |
|  | Mean (SD) | 1.23 (0.58) | 1.87 (1.06) | 1.7 (0.78) | 1.22 (0.58) | 1.25 (0.51) | 1.23 (0.53) | 1.10 (0.31) |
|  | Median (IQR) | 1 (0) | 2 (1) | 2 (1) | 1 (0) | 1 (0) | 1 (0) | 1 (0) |
|  | *p*-value | 1.00 | 0.15 | 0.31 | 0.30 | 0.80 | 0.81 | 0.07 |
| Tinnitus | Photon |  |  |  |  |  |  |  |
|  | Mean (SD) | 2.36 (1.31) | 2.19 (1.19) | 2.50 (1.08) | 2.22 (1.05) | 2.22 (1.08) | 2.26 (0.58) | 1.95 (0.92) |
|  | Median (IQR) | 2 (3) | 2 (2) | 2.5 (1) | 2 (2) | 2 (2) | 2 (2) | 2 (2) |
|  | Proton |  |  |  |  |  |  |  |
|  | Mean (SD) | 1.70 (0.96) | 1.76 (0.89) | 2.05 (1.09) | 2.11 (1.13) | 2.09 (1.15) | 2.12 (1.17) | 1.89 (1.08) |
|  | Median (IQR) | 1 (1) | 2 (1) | 2 (2) | 2 (2) | 2 (2) | 2 (2) | 2 (1) |
|  | *p*-value | *0.01 | 0.08 | *0.04 | 0.50 | 0.51 | 0.52 | 0.61 |
| Hearing difficulty | Photon |  |  |  |  |  |  |  |
|  | Mean (SD) | 2.30 (1.36) | 1.66 (1.14) | 1.85 (1.04) | 1.82 (1.12) | 1.74 (0.94) | 1.80 (0.98) | 1.47 (0.67) |
|  | Median (IQR) | 2 (2) | 1 (1) | 1 (2) | 1 (1) | 1 (1) | 1 (1) | 1 (1) |
|  | Proton |  |  |  |  |  |  |  |
|  | Mean (SD) | 1.40 (0.78) | 1.30 (0.58) | 1.60 (0.90) | 1.51 (0.97) | 1.71 (1.07) | 1.92 (1.17) | 1.63 (0.85) |
|  | Median (IQR) | 1 (1) | 1 (0) | 1 (1) | 1 (1) | 1 (1) | 1 (2) | 1 (1) |
|  | *p*-value | *<0.01 | 0.20 | 0.26 | 0.07 | 0.70 | 0.88 | 0.60 |
| Nasal blockage | Photon |  |  |  |  |  |  |  |
|  | Mean (SD) | 2.38 (1.33) | 1.86 (0.93) | 2.20 (1.14) | 2.02 (0.95) | 2.07 (1.07) | 1.73 (0.72) | 1.76 (0.83) |
|  | Median (IQR) | 2 (2) | 2 (1) | 2 (2) | 2 (1) | 2 (2) | 2 (1) | 2 (1) |
|  | Proton |  |  |  |  |  |  |  |
|  | Mean (SD) | 1.78 (1.03) | 1.50 (0.68) | 1.68 (0.89) | 1.88 (1.14) | 2.07 (1.04) | 1.97 (1.06) | 1.42 (0.59) |
|  | Median (IQR) | 1 (1) | 1 (1) | 1 (1) | 1.5 (1) | 2 (1.5) | 2 (2) | 1 (1) |
|  | *p*-value | *0.01 | 0.05 | *0.03 | 0.22 | 0.98 | 0.53 | 0.12 |
| Neck hyperpigmentation | Photon |  |  |  |  |  |  |  |
|  | Mean (SD) | 1.02 (0.16) | 1.63 (0.89) | 3.05 (1.20) | 1.94 (0.87) | 1.29 (0.60) | 1.23 (0.65) | 1.33 (0.73) |
|  | Median (IQR) | 1 (0) | 1 (1) | 3 (2) | 2 (1) | 1 (0) | 1 (0) | 1 (0) |
|  | Proton |  |  |  |  |  |  |  |
|  | Mean (SD) | 1.01 (0.12) | 1.84 (0.93) | 2.88 (1.05) | 1.95 (0.96) | 1.57 (0.75) | 1.58 (0.63) | 1.26 (0.50) |
|  | Median (IQR) | 1 (0) | 2 (1) | 3 (2) | 2 (1) | 1 (1) | 2 (1) | 1 (0) |
|  | *p*-value | 0.68 | 0.22 | 0.39 | 0.90 | 0.08 | *<0.01 | 0.95 |
| Difficulty opening mouth | Photon |  |  |  |  |  |  |  |
|  | Mean (SD) | 1.05 (0.23) | 1.86 (1.17) | 2.17 (1.42) | 1.42 (0.69) | 1.48 (0.80) | 1.26 (0.45) | 1.14 (0.47) |
|  | Median (IQR) | 1 (0) | 1 (1.5) | 2 (2) | 1 (1) | 1 (1) | 1 (1) | 1 (0) |
|  | Proton |  |  |  |  |  |  |  |
|  | Mean (SD) | 1.03 (0.17) | 1.32 (0.50) | 1.63 (0.82) | 1.22 (0.45) | 1.26 (0.52) | 1.38 (0.67) | 1.21 (0.41) |
|  | Median (IQR) | 1 (0) | 1 (1) | 1 (1) | 1 (0) | 1 (0) | 1 (1) | 1 (0) |
|  | *p*-value | 0.54 | *0.04 | 0.05 | 0.13 | 0.27 | 0.66 | 0.31 |
| Neck stiffness | Photon |  |  |  |  |  |  |  |
|  | Mean (SD) | 1.19 (0.74) | 1.36 (0.83) | 1.91 (1.21) | 1.45 (0.70) | 1.14 (0.45) | 1.11 (0.43) | 1.28 (0.71) |
|  | Median (IQR) | 1 (0) | 1 (0) | 1 (2) | 1 (1) | 1 (0) | 1 (0) | 1 (0) |
|  | Proton |  |  |  |  |  |  |  |
|  | Mean (SD) | 1.06 (0.29) | 1.33 (0.61) | 1.8 (0.85) | 1.43 (0.73) | 1.3 (0.64) | 1.48 (0.75) | 1.52 (0.72) |
|  | Median (IQR) | 1 (0) | 1 (1) | 2 (1) | 1 (1) | 1 (0) | 1 (1) | 1 (1) |
|  | *p*-value | 0.43 | 0.51 | 0.87 | 0.76 | 0.21 | *0.01 | 0.09 |

Note: 1. Symptom distress scale theoretical range 1-5, where 1 indicates “no distress at all”, 5 indicates “distressful as possible”.

2. Variables were compared using the Mann-Whitney U test for non-parametric data.

Abbreviations: IQR, Interquartile Range; RT, Radiotherapy; SD, standard deviation.

^a^ T0 refers to baseline (pre-RT period); T1 refers to 4^th^ week during RT; T2 refers to 7^th^ week during RT; T3 refers to 1 month after RT; T4 refers to 3 months after RT; T5 refers to 6 months after RT; T6 refers to 1 year after RT.

**Table S2.** Baseline characteristics of patients by radiation modality: pre- and post-IPTW adjustment

| **Characteristics** | **Before IPTW** | | | | **After IPTW** | | | |
| --- | --- | --- | --- | --- | --- | --- | --- | --- |
|  | VMAT | IMPT | Standardized  Mean  Difference | VMAT | | IMPT | Standardized  Mean  Difference |  |
| Age, mean (SD); years | 51.2 (12.1) | 49.5 (10.6) | 0.147 | 49.0 (10.6) | | 49.8 (10.7) | 0.080 |  |
| Gender;  Female  Male | 25.6%  74.4% | 21.2%  78.8% | 0.105 | 23.3%  76.7% | | 21.5%  78.5% | 0.042 |  |
| Education level (%)  High School Diploma or Less  College Degree or Higher | 79.5%  20.5% | 31.8%  68.2% | 1.094 | 60.0%  40.0% | | 52.1%  47.9% | 0.160 |  |
| Marital status; (%)  Single  Married  Divorced / Separated / Widowed | 25.0%  52.8%  22.2% | 18.5%  75.4%  6.1% | 0.550 | 22.0%  60.1%  17.9% | | 24.9%  63.7%  11.3% | 0.189 |  |
| Yearly Household Income($)(%)  < 20000  20000-33333  > 33333 | 69.2%  18.0%  12.8% | 24.2%  24.2%  51.5% | 1.091 | 45.5%  22.2%  32.3% | | 43.0%  20.5%  36.5% | 0.089 |  |
| Karnofsky Performance Status  100  90  80 | 25.0%  66.7%  8.3% | 43.1%  53.8%  3.1% | 0.424 | 27.0%  65.5%  7.7% | | 32.6%  60.4%  7.0% | 0.124 |  |
| Tumor status (%)  T1  T2  T3  T4 | 35.9%  15.4%  20.5%  28.2% | 47.0%  19.7%  21.2%  12.1% | 0.420 | 34.3%  23.0%  24.7%  17.9% | | 41.3%  18.7%  21.4%  18.6% | 0.165 |  |
| Nodal status (%)  N0  N1  N2  N3 | 18.0%  25.6%  33.3%  23.1% | 19.7%  27.3%  33.3%  19.7% | 0.089 | 11.1%  24.7%  34.0%  30.2% | | 14.1%  27.9%  31.3%  26.8% | 0.132 |  |
| Clinical AJCC staging (%)  I  II  III  IVa  IVb | 12.8%  12.8%  28.2%  41.0%  5.1% | 12.1%  13.6%  40.9%  27.3%  6.1% | 0.328 | 7.7%  16.6%  32.2%  38.6%  4.9% | | 8.8%  17.6%  36.1%  32.5%  5.0% | 0.132 |  |
| Induction chemotherapy (%) | 89.7% | 86.4% | 0.104 | 93.7% | | 90.4% | 0.122 |  |
| Treatment regimen; *n* (%)  RT alone  Chemoradiotherapy | 7.7%  92.3% | 10.6%  89.4% | 0.101 | 6.3%  93.7% | | 7.7%  92.3% | 0.055 |  |

Abbreviations: AJCC, American Joint Committee on Cancer; IMPT, Intensity-modulated proton therapy; RT, Radiotherapy. VMAT, Volumetric Modulated Arc Therapy

**Table S3.** Baseline characteristics of non-metastatic patients completing all first four consecutive surveys, by radiation modality

| **Participant characteristics** | **VMAT (n=32)** | **IMPT (n=54)** | ***p* value^a^** |
| --- | --- | --- | --- |
| Age at RT, median (IQR); years | 52.0 (43.5-60.0) | 47.5 (43.0-58.0) | 0.43 |
| Gender; *n* (%)  Female  Male | 8 (25.0)  24 (75.0) | 12 (22.2)  42 (77.8) | 0.76 |
| Education level; *n* (%)  High School Diploma or Less  College Degree or Higher | 27 (84.4)  5 (15.6) | 18 (33.3)  36 (66.7) | <0.01 |
| Marital status; *n* (%)  Single  Married  Divorced / Separated / Widowed | 5 (15.6)  19 (59.4)  8 (25.0) | 11 (20.4)  39 (72.2)  4 (07.4) | 0.07 |
| Yearly Household Income($); *n* (%)  < 20000  20000-33333  > 33333 | 20 (62.5)  7 (21.9)  5 (15.6) | 13 (24.1)  13 (24.1)  28 (51.8) | <0.01 |
| Karnofsky Performance Status  100  90  80 | 7 (21.9)  22 (68.7)  3 (09.4) | 26 (48.2)  26 (48.2)  2 (03.7) | 0.03 |
| Tumor status; *n* (%)  T1  T2  T3  T4 | 13 (40.6)  5 (15.6)  7 (21.9)  7 (21.9) | 24 (44.4)  11 (20.4)  11 (20.4)  8 (14.8) | 0.82 |
| Nodal status; *n* (%)  N0  N1  N2  N3 | 6 (18.7)  8 (25.0)  11 (34.4)  7 (21.9) | 11 (20.4)  14 (25.9)  18 (33.3)  11 (20.4) | 0.99 |
| Clinical AJCC staging; *n* (%)  I  II  III  IVa | 4 (12.5)  5 (15.6)  10 (31.3)  13 (40.6) | 7 (13.0)  8 (14.8)  22 (40.7)  17 (31.5) | 0.80 |
| Induction chemotherapy; *n* (%) | 29 (90.6) | 47 (87.0) | 0.73 |
| Treatment regimen; *n* (%)  RT alone  Chemoradiotherapy | 3 (09.4)  29 (90.6) | 6 (11.1)  48 (88.9) | 1.00 |

Abbreviations: AJCC, American Joint Committee on Cancer; IMPT, Intensity-modulated proton therapy; RT, Radiotherapy; VMAT, Volumetric Modulated Arc Therapy

^a^ Categorical variables were assessed by χ2 test or Fisher exact test. Parametric continuous variable was compared by Student’s t-test.

**Table S4.** Interaction effects of radiation modality and time on symptom distress scale changes in non-metastatic patients after the first four consecutive surveys

| **Group and Interaction effect** | **Multivariable regression** |
| --- | --- |
| Dry mouth |  |
| Acute phase within RT *(IMPT vs. VMAT)* | -0.03 (0.23) |
| 4^th^ week during RT *(IMPT x T1^a^)* | -0.05 (0.28) |
| 7^th^ week during RT *(IMPT x T2^b^)* | -0.01 (0.28) |
| 1 month after RT *(IMPT x T3^c^)* | -0.02 (0.27) |
| Oral ulcer |  |
| Acute phase within RT *(IMPT vs. VMAT)* | *-0.51 (0.21) |
| 4^th^ week during RT *(IMPT x T1^a^)* | *-0.56 (0.28) |
| 7^th^ week during RT *(IMPT x T2^b^)* | *-0.85 (0.28) |
| 1 month after RT *(IMPT x T3^c^)* | -0.13 (0.28) |
| Difficulty opening mouth |  |
| Acute phase within RT *(IMPT vs. VMAT)* | -0.25 (0.15) |
| 4^th^ week during RT *(IMPT x T1^a^)* | -0.23 (0.20) |
| 7^th^ week during RT *(IMPT x T2^b^)* | *-0.46 (0.20) |
| 1 month after RT *(IMPT x T3^c^)* | -0.05 (0.20) |
| Tinnitus |  |
| Acute phase within RT *(IMPT vs. VMAT)* | 0.11 (0.24) |
| 4^th^ week during RT *(IMPT x T1^a^)* | -0.03 (0.27) |
| 7^th^ week during RT *(IMPT x T2^b^)* | 0.01 (0.27) |
| 1 month after RT *(IMPT x T3^c^)* | 0.34 (0.27) |
| Hearing difficulty |  |
| Acute phase within RT *(IMPT vs. VMAT)* | -0.05 (0.21) |
| 4^th^ week during RT *(IMPT x T1^a^)* | -0.11 (0.25) |
| 7^th^ week during RT *(IMPT x T2^b^)* | -0.01 (0.25) |
| 1 month after RT *(IMPT x T3^c^)* | -0.03 (0.25) |

Abbreviations: IMPT, Intensity-modulated proton therapy; RT, Radiotherapy; VMAT, Volumetric Modulated Arc Therapy.

^a^ T1 refers to 4^th^ week during RT; ^b^ T2 refers to 7^th^ week during RT; ^c^ T3 refers to 1 month after RT.
